# State capacity impact on COVID-19 mortality in Brazil

**DOI:** 10.1590/0102-311XEN171523

**Published:** 2024-07-29

**Authors:** Juliana Maria de Araújo, Marco Aurélio Marques Ferreira

**Affiliations:** 1 Universidade Federal de Viçosa, Viçosa, Brasil.

**Keywords:** COVID-19, Public Administration, Public Policy, COVID-19, Administração Pública, Política Pública, COVID-19, Administración Pública, Política Pública

## Abstract

This study addresses the State’s capacity to combat the COVID-19 pandemic and contributes to the literature on crisis management in health care. We analyzed whether the capacity level impacted the State response to COVID-19 in Brazilian healthcare regions in 2020 using a set of statistical analysis techniques and public health impact analysis, including propensity score matching (PSM). Results revealed that a low COVID-19 mortality was associated with participation in municipal health consortia, schooling level of municipal health managers and the resources allocated by the Brazilian National Program for Improvement of Access and Quality of Basic Care (PMAQ). Conversely, the number of intensive care units (ICU) and life-sustaining equipment available were associated with higher mortality, as locations with a larger population concentrated operational capacity to treat the most severe cases. In conclusion, the different levels of State capacity in health regions led to different outcomes in combating the pandemic. This reinforces the importance of discussing State capacity and crisis management, since the COVID-19 confrontation in Brazil related to the level of existing resources concerning health system capacity, bureaucratic capacity and participation in consortia for sharing inputs and ensuring the provision of health services to the population.

## Resumo

Este estudo aborda a capacidade do Estado em lidar com a pandemia da COVID-19, bem como contribuir com a literatura sobre gestão de crises no setor da saúde. Analisamos se o nível de capacidade do Estado teve impacto na resposta à COVID-19 nas regiões de saúde brasileiras em 2020. O estudo utiliza um conjunto de técnicas de análise estatística e análise de impacto na saúde pública, incluindo o método de pareamento por escore de propensão (PSM). Os achados revelaram que o menor número de óbitos por COVID-19 esteve associado à participação em consórcios intermunicipais de saúde, ao nível de escolaridade dos gestores municipais de saúde e aos recursos repassados por meio do Programa Nacional de Melhoria do Acesso e da Qualidade da Atenção Básica (PMAQ). Por outro lado, o número de unidades de terapia intensiva (UTI) e de equipamentos para manutenção da vida associou-se a um maior número de óbitos, uma vez que locais populacionais maiores concentraram capacidade operacional para atender os casos mais graves. Conclui-se, portanto, que os diferentes níveis de capacitação implementados pelo Estado nas regiões de saúde brasileiras levaram a resultados distintos no enfrentamento da pandemia. Isso reforça a importância da discussão sobre a capacitação implementada pelo Estado e a gestão de crises, destacando que o resultado do enfrentamento da COVID-19 no Brasil esteve relacionado ao nível de recursos existentes, principalmente em termos de capacidade do sistema de saúde, capacidade burocrática e associação em consórcios para compartilhar insumos e garantir a prestação de serviços de saúde à população.

## Resumen

Este estudio aborda la capacidad del Estado para hacer frente a la pandemia del COVID-19 y busca contribuir a la literatura sobre gestión de crisis en el sector salud. Se analizó si el nivel de capacidad del Estado tuvo un impacto en la respuesta al COVID-19 en las regiones sanitarias brasileñas en 2020. Este estudio utilizó un conjunto de técnicas de análisis estadísticos y análisis de impacto en la salud pública, incluido el método de pareamiento por puntaje de propensión (PSM). Los hallazgos muestran que el menor número de muertes por el COVID-19 se asoció con la participación en consorcios intermunicipales de salud, el nivel de educación de los gestores municipales de salud y los recursos transferidos por el Programa Nacional de Mejoría y Acceso de la Calidad de la Atención Básica (PMAQ). Por otro lado, la cantidad de unidades de cuidados intensivos (UCI) y de equipos de soporte vital estuvo asociada a un mayor número de muertes, ya que los lugares con mayor población concentraron la capacidad operativa para tratar los casos más graves. Se concluye que los diferentes niveles de capacitación implementados por el Estado en las regiones sanitarias brasileñas llevaron a diferentes resultados para hacer frente a la pandemia. Esto refuerza la importancia de la discusión sobre la capacitación implementada por el Estado y la gestión de crisis, destacando que la respuesta al COVID-19 en Brasil estuvo relacionada con el nivel de recursos existentes, especialmente con relación a la capacidad del sistema de salud, la capacidad burocrática y la asociación en consorcios para compartir insumos y garantizar la prestación de servicios sanitarios a la población.

## Introduction

Crisis management in health care is an important interdisciplinary field of knowledge, especially given the increased risk of pandemics due to the expansion of global flows. Despite lack of consensus in the literature on health system strengthening requirements ^1^ ^,^ ^2^, many others remain concealed such as State capacity aspects.

Studies on State capacities gained prominence on the 1970s, and have recently been applied to analyze public policy implementation in democratic and globalized environments ^3^ ^,^ ^4^. State capacity has been broadly defined as the entire set of resources and abilities a State can employ to achieve its social objectives ^5^. Empirically, these capacities can be grouped in varying ways. Pires & Gomide ^5^ classify State capacities into technical-administrative - referring to human, technological, and financial resources, as well as instruments for coordinating and evaluating policies - and political-relational - linked to the articulations between bureaucrats, State agents and non-State actors.

Overall, literature on the topic converges in three directions: organizing theoretical and methodological debates; testing the concept; and discussing the advantages, disadvantages, fragility, and strength of its measurement methods ^6^. In this article, we focus on the second dimension: testing its different components. To do so, we take the dimensions proposed by Pires & Gomide ^5^ as a priori without, however, considering them as fixed. Thus, we will disaggregate the concept into new categories according to data associations.

The COVID-19 pandemic motivated some studies into investigating the importance of State capacities in combating the new disease, whether in terms of reducing cases and deaths or minimizing social harm to citizens. In this perspective, the State capacity to implement public policies would help explain the different actions adopted by government officials and, consequently, the diverse results ^7^ ^,^ ^8^.

But State capacity may not be evenly distributed across territories, resulting in governments having different sets of resources and skills to combat the pandemic thus compromising the results of actions ^7^ ^,^ ^9^. Factors that influence the State’s ability to face crises originate from before the pandemic ^10^. Moreover, State capacities are directly affected by inequalities in tax collection, economic dynamism, population size and bureaucratic profile, among other aspects that affect service provision ^11^.

In Brazil, whose territory is large and heterogeneous, municipalities are primarily responsible for managing public services in a federative context of significant inequalities between subnational entities, especially in terms of financial resources and infrastructure ^12^ ^,^ ^13^. To minimize existing disparities, health regionalization was implemented in Brazil starting in 2011 to promote State capacities through cooperation between subnational entities to expand population access to services ^14^ ^,^ ^15^. These health regions would enable the integrated planning of care networks, combining different supply capacities and diversity of resources ^16^.

Crisis situations can increase asymmetries in resource concentration, worsening health inequities. Hence, resilient health systems and governments capable of learning from crises require preparation for such events ^2^ ^,^ ^17^. In the case of COVID-19, a research agenda that subsidizes decision-making for current and future crises based on responses from health system worldwide is necessary ^1^.

Seeking to contribute to this discussion, this article analyzes how the level of local State capacity impacted COVID-19 mortality in Brazilian health regions by considering new State capacity dimensions besides health system infrastructure, such as managers’ bureaucratic capacity, participation in intermunicipal consortia and quality of the primary health services.

Incorporating more State capacity dimensions enables the construction of bases for resilient health systems, recognizing that these are not only formed by infrastructure, but also by leadership and behavior aspects of the actors involved ^18^. In the next section we will present the data and econometric strategies used for analysis. We then move on to the results and discussions and, finally, we bring the final considerations.

## Material and methods

### Data collection

We used the 450 Brazilian health regions, covering the country’s 5,570 municipalities, as units of analysis. Health region is categorized as a continuous geographic space formed by neighboring cities that share socioeconomic and cultural characteristics, as well as communication and transport networks, defined by the States in conjunction with the municipalities ^15^. As for the number of COVID-19 deaths, data were collected from the repository *Monitoring the Number of COVID-19 Cases and Deaths in Brazil at Municipal and Federative Level* ^19^, which aggregates official data provided by the Brazilian Ministry of Health and State Health Departments also informing the confirmation date. We considered as a cut-off point the number of deaths (from confirmed cases) by health region up to the 29th week in 2020.

The time frame proposed was calculated individually for each health region, starting immediately from the confirmation of the first COVID-19 cases. Importantly, the 29th week of the pandemic fell in October 2020 for 72% of the Brazilian health regions, followed by the months of November (102 regions), December (18 regions), and September (6 regions). Regarding contamination, by the end of the 29th week, more than 5 million cases and approximately 154,000 deaths had already been reported.

This cut-off was informed by the peculiar virus transmission, which initially affected peripheral areas of capitals and metropolitan regions before spreading to municipalities in the countryside ^20^. Consequently, the peaks of contamination waves were not coincident across the Brazilian territory ^21^. Moreover, the cut-off point sought to avoid influence by the start of vaccination, with debates beginning late 2020.

To represent the capacity of health regions in facing the pandemic we include variables from the technical-administrative and political-relational aspects proposed by Pires & Gomide ^5^, encompassing different dimensions according to the literature. In the technical-administrative dimension, we considered the degree of primary health care resolution - measured by the Brazilian National Program for Improvement of Access and Quality of Basic Care (PMAQ, acronym in Portuguese) values transferred -, number of intensive care units (ICU) beds, the average schooling level of health managers, number of nurses from the Family Health Strategy, vital support equipment available, as well as financial resources transferred by the Federal Government to municipalities for direct application in combating the pandemic. PMAQ is an important public policy to ensure the quality of primary health services provided, conditioning financial resources to the performance of municipal teams in the assessments conducted.

These variables were chosen due to the theoretical and empirical debate on government performance in implementing public policies, highlighting the relevance of human and material resources ^8^ ^,^ ^22^, quality of bureaucracy ^23^ and availability of financial resources ^24^. In the political-relational dimension, we considered the percentage of municipal participation in health consortia, regarded as important mechanisms of intermunicipal cooperation since the 1990s ^25^. These consortia are crucial for sharing health inputs and services, and unlike with regions a municipality can participate in more than one consortium simultaneously.

Considering the heterogeneity of the Brazilian territory, we included control variables to enabled grouping health regions with similar characteristics, namely population size, the density of inhabitants per square kilometer, the amounts spent on the Brazilian Income Transfer Program (representing local social vulnerability), the amount of Emergency Aid received from the Federal Government, fiscal effort, the Firjan Municipal Development Index (IFDM, acronym in Portuguese), Firjan Fiscal Management Index (IFGF, acronym in Portuguese), percentage of population with access to water supply, percentage of population aged 60 or over, gross domestic product per capita, and geographic location of the health region. General IFDM and IFGF were included because they represent good aggregate indicators of human development and public management quality, covering several variables not included in this study.

We collected a cross-section with the most current data available in open databases from official Brazilian sources such as Brazilian Health Informatics Department (DATASUS, acronym in Portuguese), Brazilian Institute of Geography and Statistics (IBGE, acronym in Portuguese), Brazilian Ministry of Citizenship, Transparency Portal, Firjan Institute, Institute for Applied Economic Research (IPEA, acronym in Portuguese), and National Sanitation Information System (SNIS, acronym in Portuguese). Box 1 details the variables included.

**Box 1:** Description of the study variables.

VARIABLE	DESCRIPTION	YEAR	SOURCE
Deaths	Number of COVID-19 deaths in the health region per 10,000 inhabitants, confirmed by the end of the 29th week of the pandemic	2020	*
Pop_stratum	Population size of the health region	2020	**
Nurses	Number of Family Health Strategy and ICU nurses per 10,000 inhabitants	2020	DATASUS
ICU	Total number of beds per 10,000 inhabitants allocated to COVID-19 patients	2020	DATASUS
Equipment	Number of life-sustaining equipment per 10,000 inhabitants	2020	DATASUS
FG Transfer	Amounts (in Brazilian Real) received at the municipal level, per capita, referring to the transfer of resources from the Federal Government for combating the pandemic	2020	Brazilian Ministry of Health
PMAQ	Amounts (in Brazilian Real) per 1,000 inhabitants transferred to the municipalities in each health region. Values calculated according to the performance in the 3rd cycle of the PMAQ-AB	2019	Brazilian Ministry of Health
Consortium	Percentage of municipalities in the health region that participate in health consortia	2019	IBGE
Schooling	Average schooling level of the health manager ***	2018	IBGE
Density	Average number of inhabitants per square kilometer	2010	IBGE
Bolsa_Familia	Amounts (in Brazilian Real) spent in the Brazilian Income Transfer Program, per capita	2019	Brazilian Ministry of Citizenship
Aid	Amounts (in Brazilian Real) spent with emergency aid during the pandemic by the Federal Government and destined to the vulnerable population, per capita	2020	Transparency Portal
IFDM	Average IFDM for the health region. It represents human development, encompassing education, health, employment, and income	2018	Firjan Institute
IFGF	Average IFGF for the health region. It represents the quality of fiscal management, and consists of four indicators: autonomy, personnel expenses, liquidity, and investments	2019	Firjan Institute
Effort	Average municipal tax collection effort (in %) for the health region. Represents the percentage of ISS, IPTU and ITBI revenues in relation to total revenues	2019	IPEA
Attend_Water	Average percentage of water supply service in the health region	2019	SNIS
Age60+	Average percentage of the population 60 years or older in the health region	2019	DATASUS
GDP	GDP per capita (in BRL 1,000.00)	2018	IBGE
Region	Region of the territory to which the health region belongs ^#^	2021	IBGE

DATASUS: Brazilian Health Informatics Department; GDP: gross domestic product; IBGE: Brazilian Institute of Geography and Statistics; ICU: intensive care unit; IFDM: Firjan Municipal Development Index; IFDM: Firjan Fiscal Management Index; IPEA: Institute for Applied Economic Research; IPTU: Urban Property and Territorial Tax; ISS: Services Tax; ITBI: Real Estate Transfer Tax; PMAQ: Brazilian National Program for Improvement of Access and Quality of Basic Care; SNIS: National Sanitation Information System.

Source: prepared by the authors.

* Data on the number of deaths was collected from the website *Monitoring the Number of COVID-19 Cases and Deaths in Brazil at Municipal and Federative Units Level* ^53^, which combines information from the Brazilian Ministry of Health and the State Health Departments;

** A value of 1 was assigned for health regions with up to 100,000 inhabitants, 2 for a population between 100,001 and 150,000; 3 for population between 150,001 and 250,000; 4 for population between 250,001 and 350,000; 5 for population between 350,001 and 500,000; 6 for populations between 500,001 and 1,000,000; and 7 for populations above 1,000,000 inhabitants;

*** Values assigned: 1 for incomplete primary education, 2 for complete primary education, 3 for incomplete secondary education, 4 for complete secondary education, 5 for incomplete tertiary education, 6 for complete tertiary education, 7 for specialization, 8 for master’s degree; and 9 for PhD degree;

^#^ Values assigned: 1 for Southeast, 2 for South, 3 for Central-Western, 4 for North, and 5 for Northeast.

Variables referring to equipment, human resources, and financial resources (ICUs, equipment, nurses and financial transfers) were collected only up to the 29th week of the pandemic, thus avoiding the inclusion of resources made available after the cut-off. For the Emergency Aid amounts, given the nature of the payment model, we considered the amount made available to all health regions until October 2020, which represented the 29th week for most municipalities (72%).

### Statistical analysis

ANOVA tests were performed after descriptive statistics analysis to find associations between the data and the health region’s population size. We submitted the variables to an exploratory factor analysis (EFA) to explore existing factors (i.e., latent constructs or dimensions) in the data. We used principal component analysis (PCA) for factor extraction and varimax criteria for rotation. Adequacy of the EFA to the data was verified by Kaiser-Meyer-Olkin (KMO) statistics and Bartlett’s sphericity test ^26^.

EFA allowed to explore new factors, thus justifying not limiting the nomenclatures used to those fully explored in public policies. Data characteristics also underline this choice, since EFA will aggregate them according to their respective correlation, which may not correspond to the dimensions explored in state capacity theory.

Factors related to state capacity and local characteristics were identified, the latter being important for matching the health regions, and we separated the treated and untreated groups before applying the propensity score matching (PSM) technique. This allowed us to analyze how the levels of State capacities impacted COVID-19 mortality.

As State capacities may exist to a greater or lesser degree in each locality, it would be impossible to identify treated and untreated groups by the simple existence (or not) of such capacities. We thus used the median as a separation factor between treated and untreated groups, allowing for a balanced number of observations in each group. We attributed a value of 1 (treated group) to values equal to or greater than the median, representing regions that previously invested in their State capacity, and 0 to the others (untreated group). Differences between group averages was verified using t-tests (parametric data) and the Mann-Whitney’s test (nonparametric data) ^27^.

With the groups defined, we imputed the local characteristics in the PSM to match treated and untreated groups. PSM calculated the probability of receiving treatment, interpreted as a higher level of State capacity. Matching was performed considering health regions of treated and untreated groups that presented similar probabilities according to predefined criteria ^28^.

According to Rosenbaum & Rubin ^29^ and Becker & Ichino ^30^, the probability or propensity score is the conditional probability of receiving treatment based on a set of predetermined observable variables. Since the treated and untreated groups share the same observable characteristics ^31^, the average difference in COVID-19 mortality gives us the estimated impact of State capacities. This analysis points out the contributing factors to reduce the number of COVID-19 deaths.

## Results

Table 1 details the average, standard deviation, maximum and minimum values of the descriptive statistics analysis.

**Table 1:** Descriptive statistics.

Variable	Average	SD	Lowest	Higher
Deaths	5.84	4.18	0.31	46.31
Bolsa_Familia	189.66	144.97	4.96	618.97
Aid	1,137.11	295.35	66.6	2594
IFGF	0.44	0.14	0.11	0.78
IFDM	0.66	0.08	0.44	0.83
Attend_Water	69.16	17.72	11.25	100.00
Age60+	14.56	3.71	5.07	26.52
GDP	25.72	14.62	6.34	90.06
Effort	5.57	4.82	0.57	48.83
Nurses	2.75	0.88	0.55	5.22
Consortium	56.90	40.56	0.00	100.00
PMAQ	1,160.31	782.96	8.35	4736.85
Pop_stratum	3.78	1.73	1.00	7.00
Density	147.09	571.02	0.61	7387.69
Schooling	6.09	0.51	4.56	8.00
FG Transfer	107.76	47.37	0.00	376.46
ICU	0.88	0.63	0.00	4.18
Equipment	26.77	18.83	1.43	95.35

SD: standard deviaiton.

Source: prepared by the authors.

The five factors identified by EFA and their corresponding variables are shown in Table 2. Together, these five factors had an explained percentage of variance of 73.58%, higher than the 60% recommended by the literature ^26^, showing a good explanatory power with this number of constructs. KMO statistics presented a value of 0.876, indicating a good adequacy of the data to the method, also corroborated by Bartlett’s sphericity test, which showed a sufficient degree of correlations at 1% significance.

**Table 2:** Exploratory factor analysis results for local context (factor 1) and State capacities (factors 2 to 5).

Variable	Factor 1: Local context	Factor 2: Health care system	Factor 3: Territorial bureaucracy	Factor 4: Financial	Factor 5: Operational	Communality
Region	-0.7089					0.7441
Bolsa_Familia	-0.8300					0.8620
Aid	-0.7841					0.7792
IFGF	0.8452					0.7683
IFDM	0.8872					0.8512
Attend_Water	0.6976					0.5398
Age60+	0.6156					0.7120
GDP	0.7740					0.7282
Effort	0.6159					0.8309
Nurses		0.6677				0.7061
Consortium		0.6675				0.6922
PMAQ		0.8060				0.7635
Pop_stratum			0.4748			0.6390
Density			0.7642			0.6566
Schooling			0.7218			0.5860
FG Transfer				0.8899		0.8399
ICU					0.8505	0.7797
Equipment					0.6477	0.7661

Source: prepared by the authors.

As EFA grouped variables related to the health region’s characteristics (management quality, social vulnerability, socioeconomic status, human development, location) in the first factor, we labeled it as the local context. This factor also encompassed risk behaviors related to economic inequality and, consequently, the ability to maintain stricter standards of compliance with social distancing ^28^. The literature highlights the local context as an element that conditions government results in confronting the pandemic, covering aspects such as average schooling level, location, socioeconomic condition, average population age, among others ^32^.

Disparity in these indices hinders combating health crises as local characteristics also influence the quality and quantity of resources available. Structural, this disparity impacts the federal coordination capacity to implement policies ^33^.

The second factor consisted of the health system’s general characteristics, involving the quantity of human resources, the political capacity to form consortia and the quality of primary health care. Health manager’s schooling level, population size and demographic density were grouped into the third factor, labeled territorial bureaucratic capacity. This correlation may be explained by the concentration of higher education institutions in larger and denser locations ^31^, which justifies the existence of managers with a higher level of education.

Financial resources allocated to municipalities by the Federal Government for combating the pandemic makes up the financial capacity factor, representing the mobilization of short-term resources to fight the pandemic. Finally, the fourth factor encompasses the ICU beds for COVID-19 patients and the number of life support equipment representing the operational capacity, an element of paramount importance to ensure timely care for the most severe cases.

After determining the State capacity factors, we performed the PSM to separate between treated and untreated groups. We attribute 1 to values greater than or equal to the median of each variable to form the treated group, and 0 to the others, forming the untreated group. Using the median as a separation factor allowed for a similar number of health regions in each group (Table 3). Table 3 shows the discrepancies between group averages, whose differences are statistically significant by the t-tests (parametric variables) or Mann-Whitney (nonparametric variables). We therefore confirmed that the averages in state capacities of the treated groups are superior to those of the untreated groups.

**Table 3:** Characterization of treated and untreated groups.

Dimension/Variable	Treated group		Untreated group		Difference between averages	Significance
	Observations	Average	Observations	Average		
Health system capacity						
Nurses	226	3.46	224	2.03	1.42	0.000
Consortium	225	92.94	225	20.86	72.07	0.000
PMAQ	225	1,763.40	225	557.21	1,206.19	0.000
Financial capability						
FG Transfer	225	145.36	225	70.16	75.19	0.000
Operational capacity						
ICU	227	1.36	223	0.39	0.97	0.000
Equipment	225	41.56	225	11.97	29.59	0.000
Territorial bureaucratic capacity						
Schooling	228	6.48	222	5.68	0.79	0.000
Pop_stratum	232	5.19	218	2.27	2.91	0.000
Density	225	277.89	225	16.28	261.61	0.000

Source: prepared by the authors.

After confirming the average differences, we applied the PSM to analyze the impact of State capacities on the number of registered COVID-19 deaths. We used the local context variables (factor 1) to calculate the probability of receiving treatment, matching between elements of the treated and untreated groups using the nearest neighbor criterion (i.e., similarity of probability). As shown in Table 4, the average difference in mortality between similar elements in these groups determines the impact (average treatment effect).

**Table 4:** Average treatment effect on the number of deaths using propensity score matching.

Dimension/Variable	Coefficient	SD	Significance
Matching criterion: nearest neighbor			
Health system capacity			
Nurses	-0.490	0.460	0.287
Consortium	-0.816	0.468	0.082 *
PMAQ	-1.350	0.403	0.001 **
Financial capability			
FG Transfer	0.799	0.611	0.192
Operational capacity			
ICU	0.849	0.384	0.027 ***
Equipment	0.855	0.422	0.043 ***
Territorial bureaucratic capacity			
Schooling	-0.686	0.392	0.080 *
Pop_stratum	0.731	0.366	0.046 ***
Density	-0.157	0.869	0.856
Matching criterion: second nearest neighbor			
Health system capacity			
Nurses	-0.582	0.378	0.124
Consortium	-0.728	0.451	0.107
PMAQ	-1.223	0.406	0.003 **
Financial capability			
FG Transfer	0.702	0.489	0.152
Operational capacity			
ICU	1.019	0.368	0.006 **
Equipment	1.034	0.775	0.182
Territorial bureaucratic capacity			
Schooling	-0.667	0.380	0.079 *
Pop_stratum	0.602	0.395	0.128
Density	-0.560	0.735	0.446
Matching criterion: 0.2 caliper			
Health system capacity			
Nurses	-0.490	0.460	0.287
Consortium	-0.816	0.468	0.082 *
PMAQ	-1.350	0.403	0.001 **
Financial capability			
FG Transfer	0.799	0.611	0.192
Operational capacity			
ICU	0.849	0.384	0.027 **
Equipment	0.855	0.422	0.043 ***
Territorial bureaucratic capacity			
Schooling	-0.686	0.392	0.080 *
Pop_stratum	0.731	0.366	0.046 ***
Density	-0.157	0.869	0.856

SD: standard deviation.

Source: prepared by the authors.

* Significant at 10%;

** Significant at 1%;

*** Significant at 5%.

From Table 4, we highlight the negative impact observed regarding mortality in relation to participation in health consortia, bureaucratic capacity, represented by health manager’s schooling, and PMAQ resources. These observations are important for defining coping strategies for health crises such as COVID-19. We will discuss these results in more detail below.

## Discussion

Core data characteristics (Box 1) and their great heterogeneity, revealed by the high standard deviation values observed, show a scenario of marked social and economic inequalities between health regions especially in the number of ICU beds, an essential resource for treating the most severe COVID-19 cases.

In the period analyzed, the average number of ICU-COVID beds was 0.88 per 10,000 inhabitants, below the minimum of one bed recommended by the World Health Organization ^8^. Such a situation becomes more serious when considering that 49 health regions lacked any specific ICU bed for COVID-19 patients, demonstrating a fragility of the health infrastructure before the pandemic. When considering the total number of beds, 72% of the health regions provided a lower number than recommended even for routine use, as observed by Rache et al. ^34^. These findings confirm that health systems are often unprepared to respond quickly to population demands in a pandemic context ^1^.

ANOVA testing provided an important picture of the human resources distribution and infrastructure of the Brazilian health system. Health regions serving a higher population size concentrated a greater number of ICU-COVID beds, more life maintenance equipment, as well as health managers with a higher schooling level. Conversely, regions with fewer inhabitants despite having lower infrastructure, presented a greater number of nurses, greater participation in health consortia and more PMAQ transfers, indicating better quality of primary care services.

Hence, despite a 10-year implementation of the health regions, these lack a standardization of the resources available thus revealing the fragility of regional health planning. These findings are worrisome given the importance of health regions as bases for planning care networks and privileged spaces for articulating health actions ^16^.

As health regions are centers for health planning, the primary and secondary care systems should act together to reduce patient referral to intensive care ^35^. However, larger health regions concentrated the intensive care infrastructure for the most severe COVID-19 cases, while the smaller regions showed greater investment in primary health care.

At the municipal level, Cardoso et al. ^36^ found that the income transfer policy of the Brazilian Federal Government disregarded local vulnerability, level of contamination and municipal income level. Consequently, many municipalities had to reduce their own health expenditures, as places with greater health resources received more financial transfers. New investments are therefore being made disregarding the level of preexisting resources, leading to greater planning of public policies to support governments in health crises.

Regarding impact, PSM analysis showed that operational capacity, which was concentrated in large healthcare regions, was associated with an increase in COVID-19 deaths. This may be because larger and more developed locations concentrate more people whose displacements result in greater risks of exposure. Moreover, larger locations have more opportunities to enhance State capacities to treat patients ^37^.

An increase in COVID-19 cases, even with a greater number of beds, could render the health infrastructure insufficient and lead to an increase in the number of deaths. Initially, the levels of death and contamination were directly related to population size, but following the collapse of the health system local medical-hospital structure began to directly influence the fatality rate ^8^.

Although our results highlight the importance of primary health care as a tool to halt the pandemic, the literature emphasizes other contributing aspects ^38^. In fact, the increased health system capacity’s effectiveness would depend on minimum conditions such as teams of qualified professionals, political will and financing ^39^.

PSM analysis revealed that a greater transfer of PMAQ resources is associated with a reduction in the number of COVID-19 deaths. Importantly, the PMAQ resources allocated are directly linked to the evaluation of each primary care team and Expanded Family Health and Primary Care Center (NASF-AB, acronym in Portuguese). Thus, these transfers related to the quality and resolvability of the primary care services provided. In fact, this variable had the highest coefficient in the PSM results, that is, it would have the greatest impact on reducing the number of deaths and combating the pandemic.

Primary care’s role in combating COVID-19 is founded on the need to monitor and screen infected and suspected patients, helping to control the pandemic and ensure that seriously ill patients have timely access to the healthcare system. Recent studies by Medina et al. ^40^ and Barbosa et al. ^41^ corroborate this result, demonstrating the importance of primary care to minimize health inequities, reduce infection levels, and mitigate the social and economic effects of the pandemic.

Despite the difficulty in facing crises of countries with decentralized government models, Erkoreka & Hernando‐Pérez ^42^ state that this does not pose a disadvantage. When analyzing sub-central governments in Spain, the authors found that action coordination and health system robustness, represented by financial investments, human resources, and equipment, enabled a better performance in facing the COVID-19 pandemic.

Kim & Jeong ^43^ stress that despite the importance of central governments, local actions played a crucial role in combating the pandemic. This is because in addition to national coordinated actions, local assessment allows health systems to meet population needs. Effective containment of the pandemic is related to government effectiveness ^44^, whose resources must be mobilized through political processes, determining how state capacity can and will be used ^45^. In this relational perspective, the (de)activation of State capabilities materializes, among other aspects, in the institutional arrangements formed and the interactions between different actors ^5^.

Besides primary care, we also observed a reduction in the number of deaths by greater participation in health consortia. This result corroborates Ferreira et al. ^46^, who found that municipal association via consortia was one of the most effective measures to control the pandemic.

These consortia enable health care networks to be more collaborative, allowing for the expansion of service access ^47^ and consequently the reduction of inequities in the health regions studied. The importance of municipal consortia was also discussed by Santos ^48^, who assessed their prominent role in fighting the pandemic through the provision of services, joint acquisition of inputs, personal protective and ICU equipments, promotion of educational activities, among others.

Our results also highlighted another aspect little investigated when discussing coping with COVID-19 in Brazil: the negative association between bureaucratic capacity and the number of deaths. This reinforces that the coordination of pandemic-fighting efforts based on bureaucratic capacities is an important aspect of health crisis management ^49^. Focusing on education ^50^, they identified that the manager’s schooling was important for implementing successful policies in the pandemic context, highlighting the relevance of a competent and professionalized municipal bureaucracy.

Bureaucratic capacity is widely discussed in the literature on State capacities as it relates to technical competences, that is, to the development of a competent technical team to achieve good public policy results in the most diverse areas ^51^. Thus, our results corroborate the importance of the human factor for managing crisis situations, highlighting that managers’ characteristics can influence the probability of success of the implemented actions ^52^.

## Conclusion

Our results showed that the levels of State capacity influenced COVID-19 mortality in Brazil, highlighting knowledge already acquired regarding the pandemic and reinforcing new findings. Among them, the quality of primary care emerges as a helping factor to reduce contamination levels and consequently deaths. Moreover, the results emphasized the importance of consortia as a mechanism to increase health resources for coping with crisis situations.

Association between schooling level of health managers and reduced number of COVID-19 deaths indicate the importance of bureaucratic capacity, reinforcing theoretical discussions on how bureaucratic capacity influences public policy outcomes even during health crisis.

Thus, COVID-19 mortality could be minimized by a preventive, comprehensive, and integrated approach via the articulation of actions between different entities, investment in health management training and assurance of quality health services in basic care. Finally, our results encourage debate on State capacity and their relevance and demonstrate that an effective fight against the pandemic goes beyond health system infrastructure since other capacity dimensions are equally important. As for study limitations, we emphasize that the existence of State capacity in health regions does not ensure its operation, requiring further studies to understand whether this process occurred and how it took place during the COVID-19 pandemic.

## References

[1] Gilson L, Marchal B, Ayepong I, Barasa E, Dossou JP, George A (2020). What role can health policy and systems research play in
supporting responses to COVID-19 that strengthen socially just health
systems. Health Policy Plan.

[2] Haldane V, Morgan GT (2021). From resilient to transilient health systems the deep
transformation of health systems in response to the COVID-19
pandemic. Health Policy Plan.

[3] Cingolani L The state of State capacity: a review of concepts, evidence and
measures..

[4] Gomide AA, Pereira AK, Machado R (2017). O conceito de capacidade estatal e a pesquisa
científica. Sociedade e Cultura.

[5] Pires RRC, Gomide AA (2016). Governança e capacidades estatais uma análise comparativa de
programas federais. Revista de Sociologia e Política.

[6] Souza C, Fontanelli F, Mello J, Ribeiro VMR, Lotta G, Bonamino A, Carvalho CP (2020). Implementação de políticas e atuação de gestores públicos.

[7] Ito NC, Pongeluppe LS (2020). The COVID-19 outbreak and the municipal administration responses
resource munificence, social vulnerability, and the effectiveness of public
actions. Rev Adm Pública.

[8] Pereira AK, Oliveira MS, Sampaio TS (2020). Asymmetries of state government social distancing policies in the
face of COVID-19 political and technical-administrative
aspects. Rev Adm Pública.

[9] Sanabria-Pulido P (2020). COVID-19: una prueba ácida a la capacidad de Estados, gobiernos y
sociedades. Recomendaciones de gestión y políticas públicas para una
respuesta integral y coordinada..

[10] Lima LD, Pereira AMM, Machado CV (2020). Crisis, conditioning factors, and challenges in the coordination
of Brazil's federative State in the context of COVID-19. Cad Saúde Pública.

[11] Santana MO, Gomes SC (2017). Anais do Encontro Nacional de Ensino e Pesquisa do Campo de
Públicas.

[12] Arretche M (2003). Financiamento federal e gestão local de políticas sociais o
difícil equilíbrio entre regulação, responsabilidade e
autonomia. Ciênc Saúde Colet.

[13] Santos AMSP (2008). Descentralização, desenvolvimento local e autonomia financeira
dos municípios. Quivera.

[14] Viana ALd'A, Iozzi FL (2019). Confronting health inequalities: impasses and dilemmas in the
regionalization process in Brazil.. Cad Saúde Pública.

[15] Brasil (2011). Decreto nº 7.508, de 28 de junho de 2011. Regulamenta a Lei nº
8.080, de 19 de setembro de 1990, para dispor sobre a organização do Sistema
Único de Saúde - SUS, o planejamento da saúde, a assistência à saúde e a
articulação interfederativa, e dá outras providências.. Diário Oficial da União.

[16] Lima LD, Viana ALdA, Machado CV, Albuquerque MV, Oliveira RG, Iozzi FL (2012). Regionalização e acesso à saúde nos estados brasileiros
condicionantes históricos e político-institucionais. Ciênc Saúde Colet.

[17] Duong DB, King AJ, Grépin KA, Hsu LY, Lim JF, Phillips C (2021). Strengthening national capacities for pandemic preparedness a
cross-country analysis of COVID-19 cases and deaths. Health Policy Plan.

[18] Barasa EW, Cloete K, Gilson L (2017). From bouncing back, to nurturing emergence reframing the concept
of resilience in health systems strengthening. Health Policy Plan.

[19] Cota W (2020). Monitoring the number of COVID-19 cases and deaths in Brazil at
municipal and federative units level.. SciELO Preprints.

[20] Freitas CM, Barcellos C, Villela DAM, Portela MC, Reis LC, Guimarães RM (2023). Observatório Covid-19 Fiocruz - uma análise da evolução da
pandemia de fevereiro de 2020 a abril de 2022. Ciênc Saúde Colet.

[21] Moura EC, Cortez-Escalante J, Cavalcante FV, Barreto ICHC, Sanchez MN, Santos LMP (2022). COVID-19 temporal evolution and immunization in the three
epidemiological waves, Brazil, 2020-2022. Rev Saúde Pública.

[22] Pearson CM, Mitroff II (1993). From crisis prone to crisis prepared a framework for crisis
management. The Executive.

[23] Marenco A (2017). Burocracias profissionais ampliam capacidade estatal para
implementar políticas Governos, burocratas e legislação em municípios
brasileiros. Dados Rev Ciênc Sociais.

[24] Martins DG (2021). The state of the art of institutional capacity a scoping review
of the literature in Portuguese. Cadernos EBAPE.BR.

[25] Nascimento ABFM, Fernandes ASA, Sano H, Grin EJ, Silvestre HC (2021). Cooperação intermunicipal baseada no Institutional Collective
Action os efeitos dos consórcios públicos de saúde no Brasil. Rev Adm Pública.

[26] Jr. JF Hair, Black WC, Babin BJ, Anderson RE, Tatham RL (2009). Análise multivariada de dados..

[27] Gertler PJ, Martínez S, Premand P, Rawlings LB, Vermeersch CMJ (2018). Avaliação de impacto na prática..

[28] Haupt MR, Weiss SM, Chiu M, Cuomo R, Chein JM, Mackey T (2022). Psychological and situational profiles of social distance
compliance during COVID-19. J Commun Healthc.

[29] Rosenbaum PR, Rubin DB (1983). The central role of the propensity score in observational studies
for causal effects. Biometrika.

[30] Becker SO, Ichino A (2002). Estimation of average treatment effects based on propensity
scores. Stata J.

[31] Instituto Nacional de Estudos e Pesquisas Educacionais Anísio
Teixeira Pesquisas estatísticas e indicadores educacionais..

[32] Araújo JM, Ferreira MAM (2023). A saúde em tempos de crise lições a partir da
COVID-19. Revista Katálysis.

[33] Censon D, Barcelos M (2020). O papel do estado na gestão da crise ocasionada pela COVID-19
visões distintas sobre federalismo e as relações entre União e
municípios. Revista Brasileira de Gestão e Desenvolvimento Regional.

[34] Rache B, Rocha R, Nunes L, Spinola P, Malik AM, Massuda A (2020). Necessidades de infraestrutura do SUS em preparo ao COVID19: leitos de
UTI, respiradores e ocupação hospitalar..

[35] Moreira RS (2020). COVID-19 unidades de terapia intensiva, ventiladores mecânicos e
perfis latentes de mortalidade associados à letalidade no
Brasil. Cad Saúde Pública.

[36] Cardoso RL, Azevedo RR, Pigatto JAM, Fajardo BAG, Cunha ASM (2022). Lessons from Brazil's unsuccessful fiscal decentralization policy
to fight COVID-19.. Public Adm Dev.

[37] Araújo JM, Ferreira MAM (2023). Análise das capacidades estatais no enfrentamento da pandemia da
COVID-19 no Brasil. REAd - Revista Eletrônica de Administração.

[38] Pereira AMM, Machado CV, Veny MB, Juan AMY, Recio SN (2021). Governance and State capacities against COVID-19 in Germany and
Spain national responses and health systems from a comparative
perspective. Ciênc Saúde Colet.

[39] Andrade CLT, Lima SML, Pereira CCA, Martins M, Soares FRG, Portela MC, Portela MC, Reis LGC, Lima SML (2022). COVID-19: desafios para a organização e repercussões nos sistemas e
serviços de saúde..

[40] Medina MG, Giovanella L, Bousquat A, Mendonça MHM, Aquino R, Comitê Gestor da Rede de Pesquisa em Atenção Primária à Saúde da
Abrasco (2020). Atenção primária à saúde em tempos de COVID-19 o que
fazer?. Cad Saúde Pública.

[41] Barbosa IR, Galvão MHR, Souza TA, Gomes SM, Medeiros AA, Lima KC (2020). Incidence of and mortality from COVID-19 in the older Brazilian
population and its relationship with contextual indicators an ecological
study. Rev Bras Geriatr Gerontol.

[42] Erkoreka M, Hernando-Pérez J (2022). Decentralization: a handicap in fighting the COVID-19 pandemic?
The response of the regional governments in Spain.. Public Adm Dev.

[43] Kim Y, Jeong YA (2022). The role of local governments in South Korea's COVID-19
response.. Public Adm Dev.

[44] Serikbayeva B, Abdulla K, Oskenbayev Y (2021). State capacity in responding to COVID-19. International Journal of Public Administration.

[45] Kavanagh MM, Singh R (2020). Democracy, capacity, and coercion in pandemic response COVID-19
in comparative political perspective. J Health Polit Policy Law.

[46] Ferreira MAM, Emmendoerfer M, Silvestre HMC, Correia AM (2022). Capacidade estatal e redes de cooperação pública na saúde no
controle da pandemia COVID-19. Sistemas & Gestão.

[47] Julião KS, Olivieri C (2020). Cooperação intergovernamental na política de saúde a experiência
dos consórcios públicos verticais no Ceará, Brasil. Cad Saúde Pública.

[48] Santos RJM, Santos AO, Lopes LT (2021). Planejamento e gestão.

[49] Schmidt F, Mello J, Cavalcante P (2020). Estratégias de coordenação governamental na crise da COVID-19..

[50] Lui L, Segatto CI, Albert CE, Santos RM (2023). Capacidades estatais e políticas municipais de educação durante a
pandemia de COVID-19. Cadernos Gestão Pública e Cidadania.

[51] Grin EJ (2021). O verso e o reverso da cooperação federativa e da difusão
vertical de políticas para promover capacidade estatal nos municípios
brasileiros.. Administração Pública e Gestão Social.

[52] Panos E, Dafni P, Kostas G, Zacharoula M (2009). Crisis management in the health sector qualities and
characteristics of health crisis managers. Int J Caring Sci.

[53] Cota W Monitoring the number of COVID-19 cases and deaths in Brazil at
municipal and federative units level..

